# Assessment of fine-scale resource selection and spatially explicit habitat suitability modelling for a re-introduced tiger (*Panthera tigris*) population in central India

**DOI:** 10.7717/peerj.3920

**Published:** 2017-11-03

**Authors:** Mriganka Shekhar Sarkar, Ramesh Krishnamurthy, Jeyaraj A. Johnson, Subharanjan Sen, Goutam Kumar Saha

**Affiliations:** 1 Department of Landscape Level Planning and Management, Wildlife Institute of India, Dehradun, Uttarakhand, India; 2 Department of Zoology, University of Calcutta, Kolkata, West Bengal, India; 3 Department of Habitat Ecology, Wildlife Institute of India, Dehradun, Uttarakhand, India; 4 Madhya Pradesh Forest Department, Indian Forest Service, Bhopal, Madhya Pradesh, India

**Keywords:** Tiger, Large carnivore, Reintroduction, Habitat suitability, K-select analysis, Mahalanobis D^2^, Habitat selection, Manly’s selection ratio

## Abstract

**Background:**

Large carnivores influence ecosystem functions at various scales. Thus, their local extinction is not only a species-specific conservation concern, but also reflects on the overall habitat quality and ecosystem value. Species-habitat relationships at fine scale reflect the individuals’ ability to procure resources and negotiate intraspecific competition. Such fine scale habitat choices are more pronounced in large carnivores such as tiger (*Panthera tigris*), which exhibits competitive exclusion in habitat and mate selection strategies. Although landscape level policies and conservation strategies are increasingly promoted for tiger conservation, specific management interventions require knowledge of the habitat correlates at fine scale.

**Methods:**

We studied nine radio-collared individuals of a successfully reintroduced tiger population in Panna Tiger Reserve, central India, focussing on the species-habitat relationship at fine scales. With 16 eco-geographical variables, we performed Manly’s selection ratio and K-select analyses to define population-level and individual-level variation in resource selection, respectively. We analysed the data obtained during the exploratory period of six tigers and during the settled period of eight tigers separately, and compared the consequent results. We further used the settled period characteristics to model and map habitat suitability based on the Mahalanobis D^2^ method and the Boyce index.

**Results:**

There was a clear difference in habitat selection by tigers between the exploratory and the settled period. During the exploratory period, tigers selected dense canopy and bamboo forests, but also spent time near villages and relocated village sites. However, settled tigers predominantly selected bamboo forests in complex terrain, riverine forests and teak-mixed forest, and totally avoided human settlements and agriculture areas. There were individual variations in habitat selection between exploratory and settled periods. Based on threshold limits of habitat selection by the Boyce Index, we established that 83% of core and 47% of buffer areas are now suitable habitats for tiger in this reserve.

**Discussion:**

Tiger management often focuses on large-scale measures, but this study for the first time highlights the behaviour and fine-scale individual-specific habitat selection strategies. Such knowledge is vital for management of critical tiger habitats and specifically for the success of reintroduction programs. Our spatially explicit habitat suitability map provides a baseline for conservation planning and optimizing carrying capacity of the tiger population in this reserve.

## Introduction

Large carnivores, at the apex of ecological pyramid, control ecosystem functions and ecological integrity (Ripple et al., 2014). Therefore, population declines and local extinctions of large carnivores necessitate restoration efforts to maintain ecological integrity (Beschta & Ripple, 2009; Wallach et al., 2009; Estes et al., 2011; Ripple et al., 2014). These predators face the imminent risk of declining numbers and local losses due to rapidly contracting geographic ranges, fragmented habitats (Morrison et al., 2007; Ceballos & Ehrlich, 2002; Ripple et al., 2014) and poaching. Tiger (*Panthera tigris*) is a top habitat generalist, and inhabits dry deciduous, moist deciduous, semi-evergreen, wet evergreen, riverine, swamp and mangrove ecosystems. This species is admirably resilient and can adapt to a wide spectrum of topographic and climatic conditions (Chundawat, 2011; Imam, Kushwaha & Singh, 2009). Recent estimates show a loss of 93% of tiger habitats in the last century (Dinerstein et al., 2007). The Global Tiger Initiative (GTI) envisioned doubling the existing wild tiger populations by 2,022 and identified several tiger conservation landscapes (TCL) in 13 tiger-range countries (Wikramanayake et al., 2011). Given that TCLs are characterized by unique eco-climatic factors, tiger responses to the immediate environment (fine scale variables) are not yet fully understood. Such knowledge is critical for providing specific management inputs, especially in tropical landscapes where resources are limited and/or highly dynamic (Wikramanayake et al., 2011; Walston et al., 2010; Global Tiger Initiative Secretariat, 2012).

Fine-scale habitat selection by mammals is driven by several benefits over meso-scale and landscape-scale habitat features (Goulart et al., 2009). Habitat correlates at various scales interact to shape and influence the cost of dispersal (Krishnamurthy et al., 2016) and gene flow (Reddy et al., 2017) in larger landscapes which further enhance the fitness of animals in a dynamic meta-population (Singh et al., 2017). Small-scale variations in resource distribution may not impact the home ranges of each individual, whereas landscape scale changes may require significant shifts (Morris, 1987), involving resistances at multiple scales. As the scale size increases, the animals are less likely to know the environments in which they reside or explore (Orians, 1991). When resource patch sizes are small, animals can either use all patches or continue to use some preferentially over others (Morris, 1984). This familiarity often determines the critical habitat for the species, which in turn serves as an important information for wildlife managers especially in human-dominated landscapes.

The tiger population in Panna Tiger Reserve, central India, became locally extinct in 2009. Subsequently, as part of species recovery program, six founder animals (one male and five females) were released in this reserve. These animals bred successfully and the population recovered rapidly to over 30 individuals by 2014. We modelled tiger-habitat relationships using the location data of nine of these tigers (six founders and three offspring). Habitat use was defined as tiger locations in a particular land cover class, and these were considered as different resource types. In this study, we analysed habitat selection by the reintroduced tiger population with respect to key eco-geographical features during the exploratory and subsequent settled periods. We further constructed habitat suitability maps based on fine-scale habitat selection and identified the presence of large parts of unsuitable/poor quality habitats in Panna Tiger Reserve, with implications for improving the habitat quality and carrying capacity of tigers in this reserve.

## Material and Method

### Ethics statement

This study was conducted in strict accordance with animal ethics including capture and handling as approved by the National Tiger Conservation Authority, New Delhi (the statutory body of the Government of India) and Madhya Pradesh Forest Department, Madhya Pradesh, India (MPFD Letter No./Exp./2009/1205 dated 31/8/09). This study was also approved by the institutional review board of the Wildlife Institute of India, Dehradun, India (No. WII/KR/Project/PTMP/14/2009). All human interventions such as administration of immobilizing drugs, capture, radio collaring, transport and monitoring were carried out by qualified professionals and with care to minimize adverse impacts on the reintroduced tigers and their offspring.

### Study area

**Figure 1:**
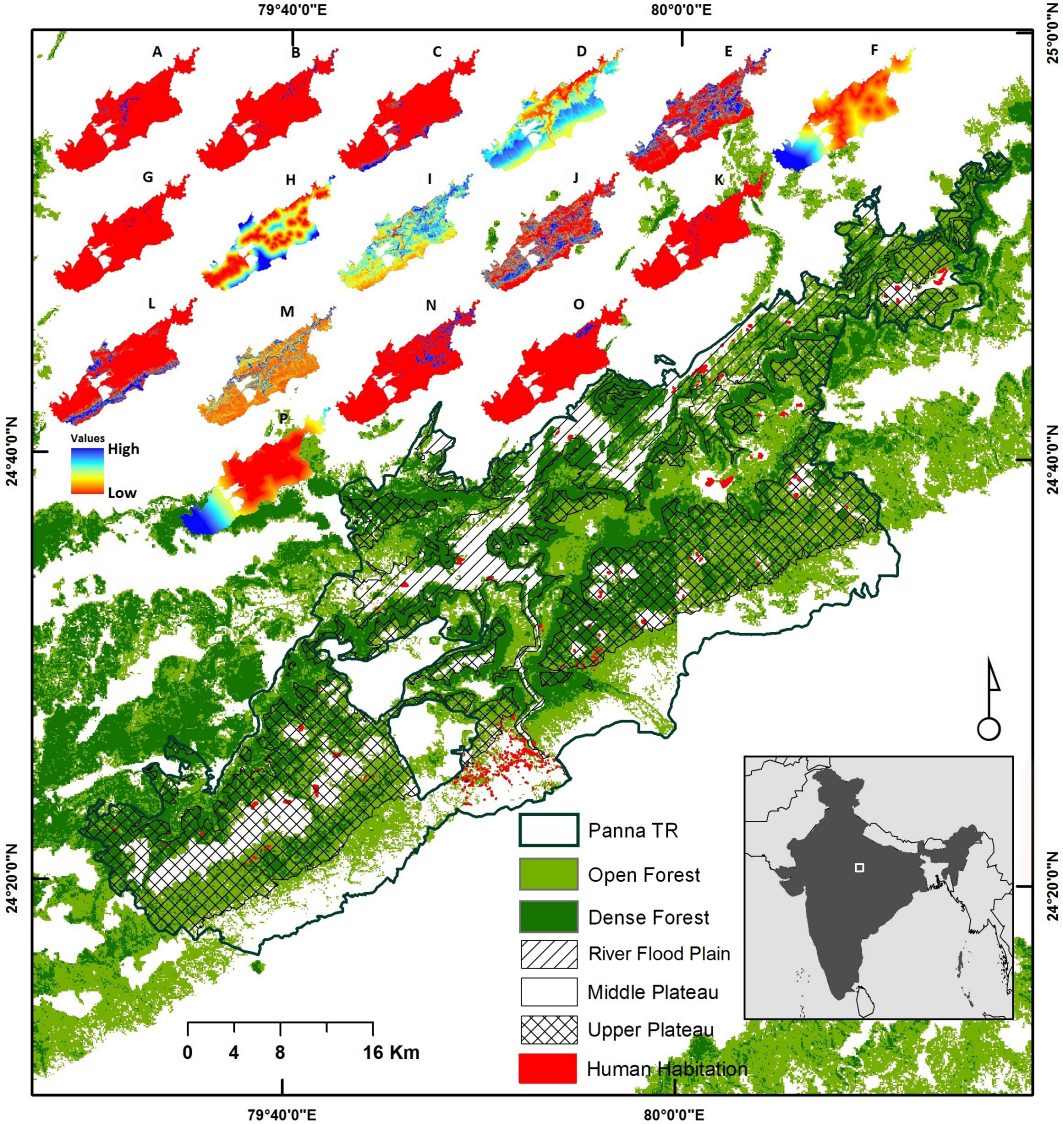
Study area map. Study area map showing geographical location of Panna Tiger Reserve (Panna TR) within India (lower inset box) and distribution of forest cover, topography, and human habitation within the reserve (key at centre bottom of panel). Upper inset maps A–P with key on the left of the panel indicate ecological and geographical variables ((A) *Anogeissus pendula* forest; (B) Bamboo mixed forest; (C) Barren land; (D) Elevation; (E) Dense mixed forest; (F) Distance to water sources; (G) Grassland; (H): Distance to human settlements; (I): Normalized Difference Vegetation Index; (J) Open mixed forest; (K) Riverine forest; (L) Scrubland; (M) Slope; (N): Teak mixed forest; (O) Pure teak forest and (P) Distance from Panna TR core area.) (Source: Remote Sensing and GIS Cell, Wildlife Institute of India).

We conducted this study in Panna Tiger Reserve (henceforth Panna TR), Madhya Pradesh, central India (Fig. 1) that largely consists of tropical dry deciduous forest landscape. Panna TR is composed of three plateaus divided by steep escarpments (50°–85°) and gorges (Fig. 1). Upper and lower plateaus are relatively flat as compared to the middle one. The middle plateau is undulating and has sparsely distributed hillocks. The lower plateau is characterized by the Ken River flood plain that flows 55 km within the reserve. With recent expansion of buffer regions, total area of the reserve is approximately 1,700 km^2^, of which the core zone comprises of 543 km^2^. Panna TR is classified as high-rainfall dry deciduous forest and largely depends on monsoon rainfall from July to September, which usually fluctuates between 600 and 1,100 mm (Jayapal, Qureshi & Chellam, 2007). This protected area is part of the Central Indian Highlands, and falls in the Vindyan Hill Range. Between 1987 and 2014, 13 villages (983 families) were voluntarily relocated from the core area to outside Panna TR, ensuring recovery of 19 km^2^ within the core area. This area has several wild prey species such as spotted deer (*Axis axis*), sambar deer (*Rusa unicolor*), wild pig (*Sus scrofa*), nilgai (*Boselaphus tragocamelus*), common langur (*Semnopithecus entellus*) and rhesus macaque (*Macaca mulatta*). This reserve also harbours a considerable number of feral cattle that were left behind by the people who were relocated.

### Location data

Between March 2009 and January 2014, six adult tigers were captured from three source populations in central India by chemical immobilization, radio-collared and released in Panna TR. These six animals, identified as T1, T2, T3, T4, T5 and T6 based on chronological order of release, represent one male (T3) and five females including two (T4 and T5) orphaned females raised in a semi-captive environment. When a translocated female tiger reproduced, cubs were named to indicate respective mother, litter number, and individual cub number, e.g., P111 denotes tiger T1’s first litter and cub number one. In addition to the founders, three of the first generation tigers (P111, P212 and P213) were also radio-collared and monitored. P111 and P212 were males whereas P213 was a female. We divided movement and occupancy patterns of founder individuals into two distinct time periods. The exploratory period was when each founder explored the area before establishing its home range, and the settled period was when each tiger occupied a distinct home range (Sarkar et al., 2016). We further assumed that the offspring included in this study had already established their home ranges, as these were radio-collared after they attained adulthood and did not show exploratory behaviour. A total of 11,077 GPS locations were obtained for these nine tigers, of which 70% locations were retrieved remotely from Argos/Iridium—GPS collars and the rest were collected by VHF receiver in the field. Detailed description of tiger locations during the study period is provided in Supplemental Information 1.

### Land cover classification

We processed 30 m spatial resolution LANDSAT-8 (November 2014) satellite imagery (Scene ID: LC81440432014330LGN00) for land cover classification of the area. A false colour composite (FCC) map was initially analysed for histogram equalization. We then used a hybrid classification method (Ruppert et al., 1997) involving unsupervised and supervised classification tools, supported by 464 ground control points to classify the area into 13 landcover classes that are relevant as tiger habitat. These classes include *Anogeissus pendula* forest, bamboo mixed forest, barren land, dense mixed forest, grassland, open mixed forest, riverine forest, scrubland, teak mixed forest, pure teak forest, wetlands and river. Land cover classes such as human settlement and agriculture land were separately digitized in high resolution Google Earth imagery and were incorporated/recoded in the final classified image using the area of interest (AOI) tool. The final image was cleaned and smoothed based on a moving window of majority values and recoding of various classes for efficient classification. An accuracy assessment of the final classified map was done based on reference locations retrieved from the high resolution Google Earth imagery and field data. The classified map was evaluated by using the kappa coefficient method, which calculates the degree of agreement between reference locations and the actual classification (Congalton, 1988). All these analyses were implemented in the ERDAS Imagine v.9.3 program (Leica Geosystems, Norcross, GA, USA). A detailed description of each land cover class and related vegetation composition within each class is provided in Table 1.

**Table 1:** Eco-geographical variables used in K-select and Mahalanobis D^2^ suitability. Description of eco-geographical variables used in K-select and Mahalanobis D^2^ suitability modelling. Variables marked with asterisk (∗) were derived from the land cover map.

Eco-geographical variable	Abbreviation	Description	Value range	Area (km^2^)
*Anogeissus pendula* forest*	AnaFor (Ap)	Proportion of *Anogeissus pendula* forest in each resource unit (RU). **Vegetation composition:** pure patches of *Anogeissus pendula* with a few patches of short length grass species.	0–1	21.17
Bamboo mixed forest*	BamMxfor (Bmf)	Proportion of Bamboo Mixed Forest in each RU. **Vegetation composition:***Dendrocalamus strictus* as major species.	0–1	188.91
Barren land*	BarLan (Bl)	Proportion of barren land vegetation in each RU. Open land with sparse distribution of small grass and thorny plant species.	0–1	88.47
Elevation	DEM (Dm)	Digital elevation data from Advanced Spaceborne Thermal Emission and Reflection Radiometer (ASTER).	166–546 (m)	–
Dense mixed forest*	DenMxFor (Dmf)	Proportion of dense vegetation in each RU. (>40% canopy density) **Vegetation composition:** Major tree species are *Terminalia arjuna, Aegle marmeloe, Buchanania lanzan, Anogeissus latifolia, Cochlospermum religiosum, Lannea grandis, Acacia catechu, Anogeissus pendula, Mitragyna parviflolia, Limonia acidissimal, Sterculia urens, Madhuca longifolia, Butea monosperma, Terminalia tomentosa, Boswella serrata, Lagerstroemia parviflora* and *Tectona grandis.*	0–1	507.78
Distance to water sources	DisWatsor (Dws)	Euclidean distance from river, major wetlands and artificial water sources.	0–30,471(m)	–
Grassland*	GrsLan (Gl)	Proportion of grassy vegetation in each RU. Area of old relocated villages. Converted to pure grassland with sparse distribution of trees (Note: most of the grasslands were formed after vegetational succession in old evacuated villages)	0–1	46.10
Distance to human settlements	DisHuSet (Dhs)	Euclidean distance from human settlement and Agricultural land	0–12,091(m)	–
Normalized Difference Vegetation Index	NDVI (Ndvi)	NDVI was calculated using red and near infrared band. NDVI calculator 9.1 was used. This is a plugin extension tool in Arc GIS 9.3.	0.19 to + 0.50	–
Open mixed forest*	OpnMxFor (Omf)	Proportion of Open vegetation in each RU. (≤40% canopy density). **Vegetation composition:** Major tree species as described in DenMxFor. *Ziziphus oenoplia*, *Ziziphus mauritiana* and *Lantana camera* are included in this group.	0–1	498.86
Riverine forest*	RivFor (Rf)	Proportion of riverine vegetation area in each RUs. Area is mainly open type vegetation. Rocky outcrop and wide sandy river bank. Vegetation composition: dominating species is *Syzygium cumini.*	0–1	11.87
Scrubland*	ScrLan (Sl)	Proportion of scrubby vegetation area in each RU. **Vegetation composition:** Sparse distribution of *Lantana camera*, *Ziziphus oenoplia*, Ziziphus *mauritiana*, small grass species and thorny plants.	0–1	216.38
Slope	Slope (Slp)	Slope in degrees calculated by DEM surface tool, a plugin extension tool in Arc GIS 9.3.	0–49.34 (Degree)	–
Teak mixed forest*	TkMxFor(Tmf)	Proportion of teak dominated mixed vegetation area in each RU. **Vegetation composition:** Teak is dominant. Other tree species as described in DenMxFor period.	0–1	98.87
Pure teak forest*	PurTk (Ptf)	Proportion of pure teak vegetation area in each RU. **Vegetation composition:***Tectona Grandis.*	0–1	17.59
Distance from Panna TR core area	DistNP (Dnp)	Euclidean distance from Panna Tiger Reserve core zone.	0–36,022 (m)	–

### Eco-geographical variables

**Figure 2:**
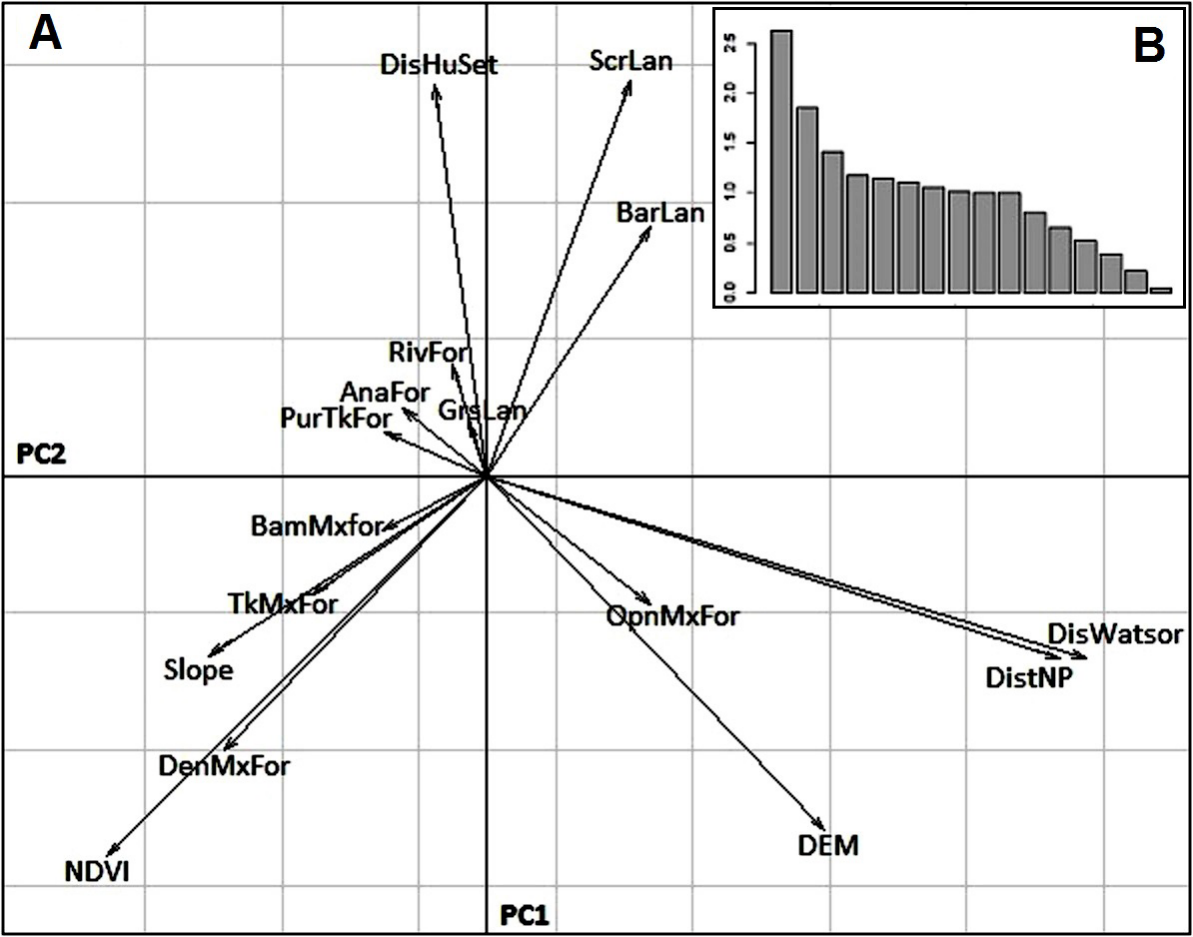
Principal component analysis. (A) Plot showing first two Principal Components describing the relationships between 16 eco-geographical variables* (EGVs) in Panna TR, Madhya Pradesh, India. (B) Bar plot of eigenvalues. (*Details of each eco-geographical variable abbreviation were provided in Table 1).

We created 16 eco-geographical (EGV) variables for K-select analysis (Calenge, Dufour & Maillard, 2005) to detect individual variation in habitat selection and to compute a Mahalanobis D^2^ model (Clark, Dunn & Smith, 1993) for predicting habitat suitability for tiger in Panna (Table 1). The study area was composed of a set of discrete resource units (RU), within which several habitat variables were measured (Manly et al., 2002). These RUs were considered as the pixels of each raster map. We tested spatial autocorrelation among all EGVs at different spatial scales (i.e., 30 m, 60 m and 90 m). The 90 m spatial scale showed minimal autocorrelation among EGVs (Orians & Wittenberger, 1991; Boyce et al., 2003) and hence allowed us to discriminate among variables and to obtain reliable results. Therefore, for fine-scale analysis, we considered each RU as a 90 m × 90 m pixel of each habitat variable, derived from 30 m spatial resolution imageries using the focal statistics tool in Arc Map v. 9.3 (ESRI, Redlands, CA, USA). The selected scale (grain) of RU was considered as the minimum space that such large mammals would require for resource procurement at a given time. Ten EGVs represented the proportion of each land cover class in each RU (i.e., 90 m cell size); while for six continuous variables, mean values of 30 m pixel EGVs were obtained for each resource unit (90 m cell size) using ArcGIS 9.3 (ESRI, Redlands, CA, USA). All 16 EGVs were subjected to pair-wise correlation analysis using Pearson correlation coefficients. Subsequently, we performed eigenvector-based multivariate analysis to perform principal component analysis on these EGVs (PCA; Fig. 2), using the default parameterization defined in the *Adehabitat* package (Calenge, 2006) in program R version 2.8.1 (R Development Core Team, 2009). This also provided an understanding of spatial configuration of the EGVs, reflecting the landscape characteristics.

### Habitat selection

#### Manly’s selection ratio

We assessed the habitat selection of tigers by comparing use and availability of habitat types at individual home range levels using type III design (Manly et al., 2002), which, in contrast to type I and type II design of habitat selection (Thomas & Taylor, 1990), yields precise estimates of habitat selection ratio (use vs. availability) at the individual level. In this method, resource use and availability were measured for each radio-collared tiger. Given that all the resources are not equally available to all tigers, the availability weights vary among individuals. We calculated 100% minimum convex polygons (MCP) for all tigers during their exploratory as well as settled periods, because the MCP home range provides a temporal snapshot of space use by tigers. Habitat preference was computed for each individual tiger and then these statistics were summed to obtain Manly’s global selection ratio during exploratory and settled periods. We also separately computed habitat selection for each individual from both sex during their settlement period. For each radio-collared tiger, the available resource type corresponded to the pixels falling inside the limits of the 100% MCP enclosing all its locations (these available resources are therefore specific to each animal). The used resources comprised of all the pixels corresponded to tiger-use locations, and the utilization weights corresponded to the proportion (magnitude) of locations falling in each resource type. We calculated Manly’s global selection ratios and *χ*^2^ values to estimate the overall deviation from random use of habitat types in program R version 2.8.1 (R Development Core Team, 2009) using the *AdehabitatHS* package (Calenge, 2006). Manly’s global selection ratios consists of computing each *χ*^2^ value per animal, and then summing these statistics to obtain a global measure of the habitat selection.

#### K-select analysis

We used K-select analysis (Calenge, Dufour & Maillard, 2005) to compute categories of animals with similar patterns of habitat selection (Calenge, Dufour & Maillard, 2005; Calenge, 2007; Hansen et al., 2009a; Hansen et al., 2009b). This robust analysis allowed us to include a large number of both categorical and continuous variables in the analysis and to take into account minute differences in individual habitat selection (Calenge, Dufour & Maillard, 2005; Calenge, 2007). We focused on marginality (Hausser, 1995; Dolédec, Chessel & Gimaret-Carpentier, 2000; Hirzel et al., 2002), a standard that measures the squared Euclidean distance between the average habitat conditions used by a tiger and the average habitat conditions available to it. In this method, a larger value of marginality shows stronger habitat selection. There are numerous internal (i.e., age and sex) and external factors (i.e., habitat availability) that potentially influence habitat selection and tend to increase variability. Averaging selection ratios without investigating variability is not appropriate, and, therefore, we employed an Eigen analysis (Calenge & Dufour, 2006) of the marginality vectors to summarize habitat selection common to all radio-collared tigers used in this analysis. An *a priori* randomization test was performed between the exploratory and the settled periods to determine individual-specific variations in habitat selection and also to establish a non-random process in the selection (Manly, 1997). This test was achieved by considering equi-probability of random allocation of RUs available to a particular tiger and by re-computing marginality for randomized data sets (Manly, 1997; Calenge, Dufour & Maillard, 2005). Observed marginality was then compared to randomized values of marginality to determine whether selection was significant for this set of variables. These randomized values were drawn from 10,000 sets of localizations distributed over the study area, thus enabling quantification of habitat availability in the study area to be compared against used locations. All these analyses were carried out with the *AdehabitatHS* package (Calenge, 2006; Calenge, 2011) in program R version 2.8.1 (R Development Core Team, 2009).

**Table 2:** Results of randomization tests. Results of randomization tests for K-select analysis of habitat selection by tigers during the exploratory and the settled periods.

	T1	T2	T3	T4	T5	T6	P111	P212	P213
**Exploratory period**									
Tests of the marginality (Bonferroni *α* level = 0.05/6 = 0.0083)									
Individual observed marginality value	0.81	1.66	1.44	1.14	4.39	2.96	–	–	–
*P*-value	0.000099	0.000099	0.000099	0.000099	0.000099	0.000099	–	–	–
**Settled Period**									
Tests of the marginality (Bonferroni *α* level = 0.05/8 = 0.0062)									
Individual observed marginality value	0.54	0.33	0.91	1.20	1.56	–	0.91	0.43	0.89
*P*-value	0.005	0.020^*^	0.0002	0.0003	0.0002	–	0.0006	0.011^*^	0.0008

**Notes.**

* Asterisk (*) indicates non-significant at the 5% *α* level but significant at the 10% *α* level.

### Habitat suitability

We used Mahalanobis distance (D^2^) statistics (Clark, Dunn & Smith, 1993) to generate a habitat suitability map of reintroduced tigers in Panna TR (core and buffer). This was a measure of dissimilarity between average habitat characteristics at each RU (90 m × 90 m pixel) and the mean of habitat characteristics estimated from cumulative tiger locations derived particularly during their settled period. Habitat quality is inversely proportional to D^2^. D^2^ has a chi-square distribution with n degrees of freedom (*n* = number of EGVs), assuming multivariate normality. The function *mahasub* in the AdehabitatHS package, (Calenge, 2006) was used to compute a map with a continuous gradient of suitability (pixels represented by *p*-values ranging between 0 and 1) from D^2^. Although this gradient of suitability will represent wide variation, it would be more meaningful from a management perspective to visualize entire forests in a few classes (e.g., optimal, suitable, marginal and unsuitable/poor quality). It is also noteworthy that a continuous scale is often misleading for habitat management, because the suitability index may not be linearly proportional to probability of use in a real environment (Hirzel et al., 2006). Real curves may show steps or exponential patterns. Therefore, a curve of the ratio of expected to predicted frequencies of evaluations points could give more meaningful insights (Boyce et al., 2002; Fielding & Bell, 1997). This curve provides the accuracy of the habitat suitability map as well as an objective criterion for choosing thresholds for reclassifying suitability maps into a few classes. Here we used all 16 EGVs (Table 2) to compute the habitat suitability map, which was further classified into 20 classes (with 0.05 probability intervals), and further calculated predicted-to-expected ratios (*Fi*) for each class (Hirzel et al., 2006):

(1) \begin{eqnarray*}{F}_{i}={p}_{i}/{E}_{i}\end{eqnarray*}

where *p*_*i*_ is the predicted frequency of evaluation points in class *i*, and *E*_*i*_ is the expected frequency, expressed as relative area covered by each class. We plotted *E*_*i*_ against class intervals (Calenge, 2006) and reclassified the suitability map into two broad classes (Poor or Unsuitable, and Suitable) by choosing threshold points from the *F*_*i*_ curve. *F*_*i*_ = 1 indicates a random model when presences are equal to expected by chance. We chose this point as the boundary between unsuitable (*F*_*i*_ < 1) and suitable (*F*_*i*_ > 1) habitats (Hirzel et al., 2006). Accuracy of the predicted habitat suitability map was evaluated by the Boyce Index (Hirzel et al., 2006; Boyce et al., 2002), wherein zero indicates random effect; negative value indicates avoidance or non-suitable/poor quality habitats and positive values are linearly related to level of suitability, i.e., higher positive value means higher suitability. Accordingly, the *boyce* function in package *ENiRG* (Cánovas et al., 2016) in program R version 2.8.1 (R Development Core Team, 2009) was used to reclassify the habitat suitability map. Prior to K select analysis and Mahalanobish D^2^, we corrected for spatial autocorrelation in the spatial patterns of location data to avoid over-estimation of predictive performance (Merckx et al., 2011; Phillips et al., 2009; Veloz, 2009). All duplicate occurrence locations were removed. Similar to Vicente et al. (2013), aggregations in animal occurrences were manually removed by selecting a subset of the original occurrences with a minimal distance optimizing the aggregation index (Clark & Evans, 1954). A value of *R* = 1 indicates that the points are randomly distributed; *R* > 1 suggests ordering; while *R* < 1 suggests clustering. For each animal, we removed all records from the closest neighbour from the original dataset of the settled period. We performed repeated iterations until the aggregation index reached *R* = 1 for each animal. This ensured that the pattern of locations was no longer autocorrelated. The computation of aggression index was carried out using the ‘*spatstat*’ package in R (Baddeley & Turner, 2005; Baddeley & Turner, 2015). After correcting for spatial autocorrelation, we obtained a sum of 2,570 locations for all settled animals, which were finally used for K-select analysis and Mahalanobish D^2^ habitat suitability modelling.

## Results

### Habitat composition

The classified land cover map of Panna TR had an overall accuracy of 88.8% with a Kappa coefficient value of 0.80. The habitat is dominated by pure teak forest on the lower plateau and slopes and covering 1.03% of total area. The middle and lower plateaus have pure *Anogeissus pendula* forests and grasslands, which cover 1.25% and 2.71% of the area, respectively. Along the river, 0.70% of the landscape is riverine forest. Most of the forest cover on the middle and lower plateaus is dense mixed forest, open mixed forest and teak mixed forest accounting for 29.87%, 29.34% and 5.82% respectively, while the barren and scrublands fall along the southern part of the upper plateau and cover 5.20% and 12.73% of entire buffer region, respectively. All 16 EGVs weakly correlated with each other and therefore all EGVs were used for further analyses (Supplemental Information 2). From the projection of EGVs on the factor plane, the 1st principal component explained 24% of total variance among variables. Barren land and scrubland were positively correlated with each other whereas both were negatively correlated with dense mixed forest, teak mixed forest and NDVI. Higher values of slope (hilly regions and escarpments) and dense vegetation were correlated with high value of NDVI and bamboo mixed forest (Fig. 2). The second principal component, which explained 12% of the variation, showed that higher elevation or upper plateau is mostly associated with open mixed forest. Pure teak forest, grassland and *Anogeissus pendula* forest were negatively correlated with open mix forest, since they are found mostly at the flat area around the river (Fig. 2).

**Figure 3:**
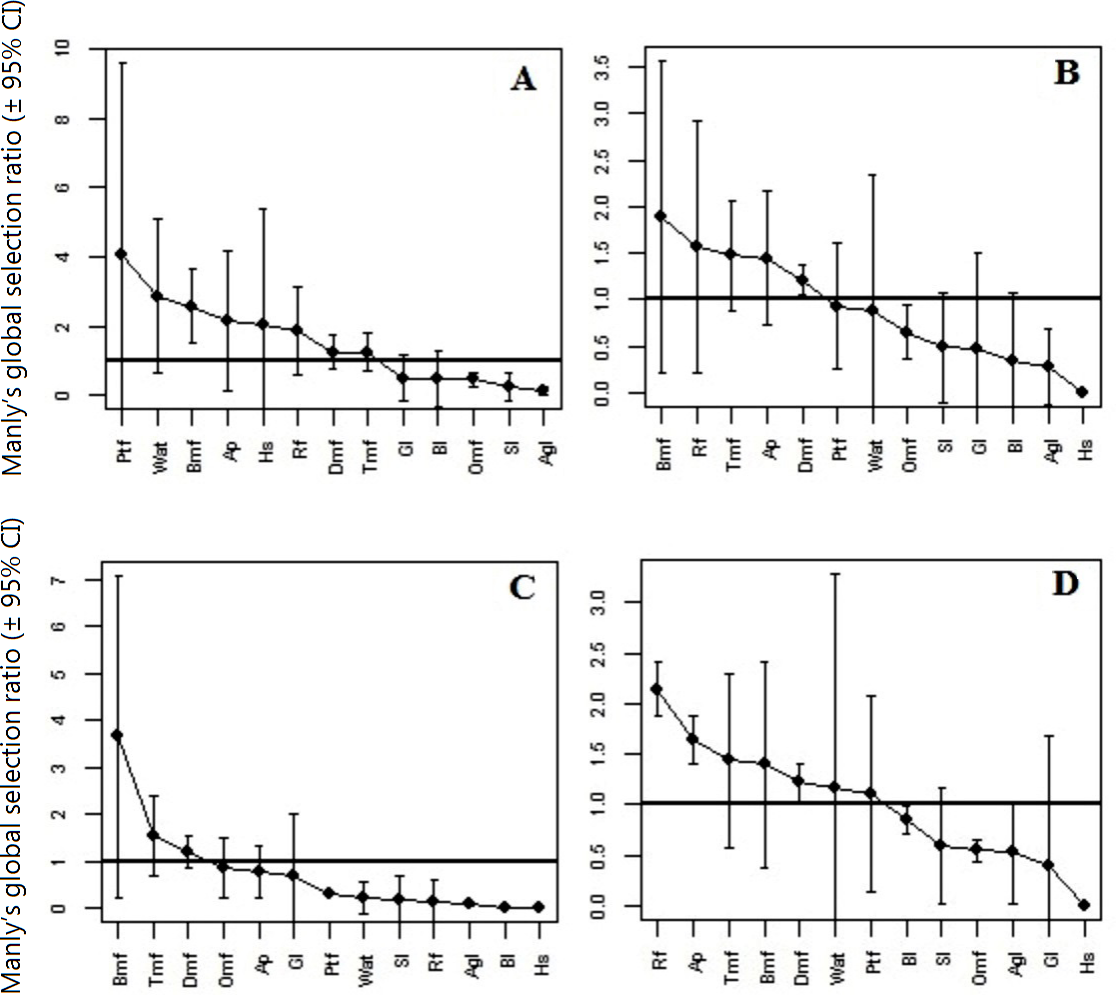
Manly’s selection ratio. Manly’s selection ratio of different land cover classes in Panna Tiger Reserve, Madhya Pradesh, India. ((A) Habitat preference of tigers during the exploratory period; (B) Habitat preference of tigers during the settled period; (C) Habitat preference of male tigers during the settled period and (D): Habitat preference of female tigers during the settled period.) (A–D) represent Global Manly’s Selection ratio with ±95% confidence intervals (CI) of the habitat types analysed in each category. Black dots (•) represent the mean selectivity rate of each habitat type. A habitat type can be considered as avoided if the global selection ratio is located in the 0–1 interval, while it can be considered positively selected if the value is larger than 1. Note differences in ordinate scale among (A–D). (Refer to Table 1 for a detailed descriptions of habitat variable abbreviations).

### Habitat selection

#### Manly’s selection ratio

Patterns of habitat selection by reintroduced tigers and their offspring significantly deviated from random during the exploratory (χ^2^ = *α*, *df* = 66, *p* < 0.0001) as well as the settled period (*χ*^2^ = 887.63, *df* = 51, *p* < 0.0001), and showed clear contrast between the exploratory and the settled periods (Figs. 3A and 3B). During the exploratory period, pure teak forest, *Anogeissus pendula* and water sources such as river and small wetlands were highly preferred by all reintroduced tigers. This was also the period when most of the tigers preferred riverine forests and water sources, especially man-made water bodies closer to village areas. However, during the settled period, animals appeared to have understood the landscape configuration and avoided disturbance zones, choosing natural water resources and other areas free of human activities. Accordingly, the habitat preferences of tigers were bamboo mixed forest >riverine forest >teak mixed forest >*Anogeissus pendula* forest >dense mixed forest. Bamboo mixed forest was highly preferred by all reintroduced tigers and this particular land cover type occurs mostly along the escarpment, which is characterized by complex terrain features and is devoid of human disturbance. Habitat selection by males and females also had significant deviation from random during settled period (χ^2^ = 324.51, *df* = 21, *p* < 0.0001 and *χ*^2^ = 563.12, *df* = 30, *p* < 0.0001, respectively) and the sex specific habitat choice was apparent (Figs.  3C & 3D). Males preferred bamboo mixed forest followed by teak mixed and dense mixed forest, whereas females preferred riverine forest followed by *Anogeissus pendula* forest, bamboo mixed forest and dense mixed forest. All settled tigers avoided human settlements within and adjoining the national park.

#### K-select analysis

**Figure 4:**
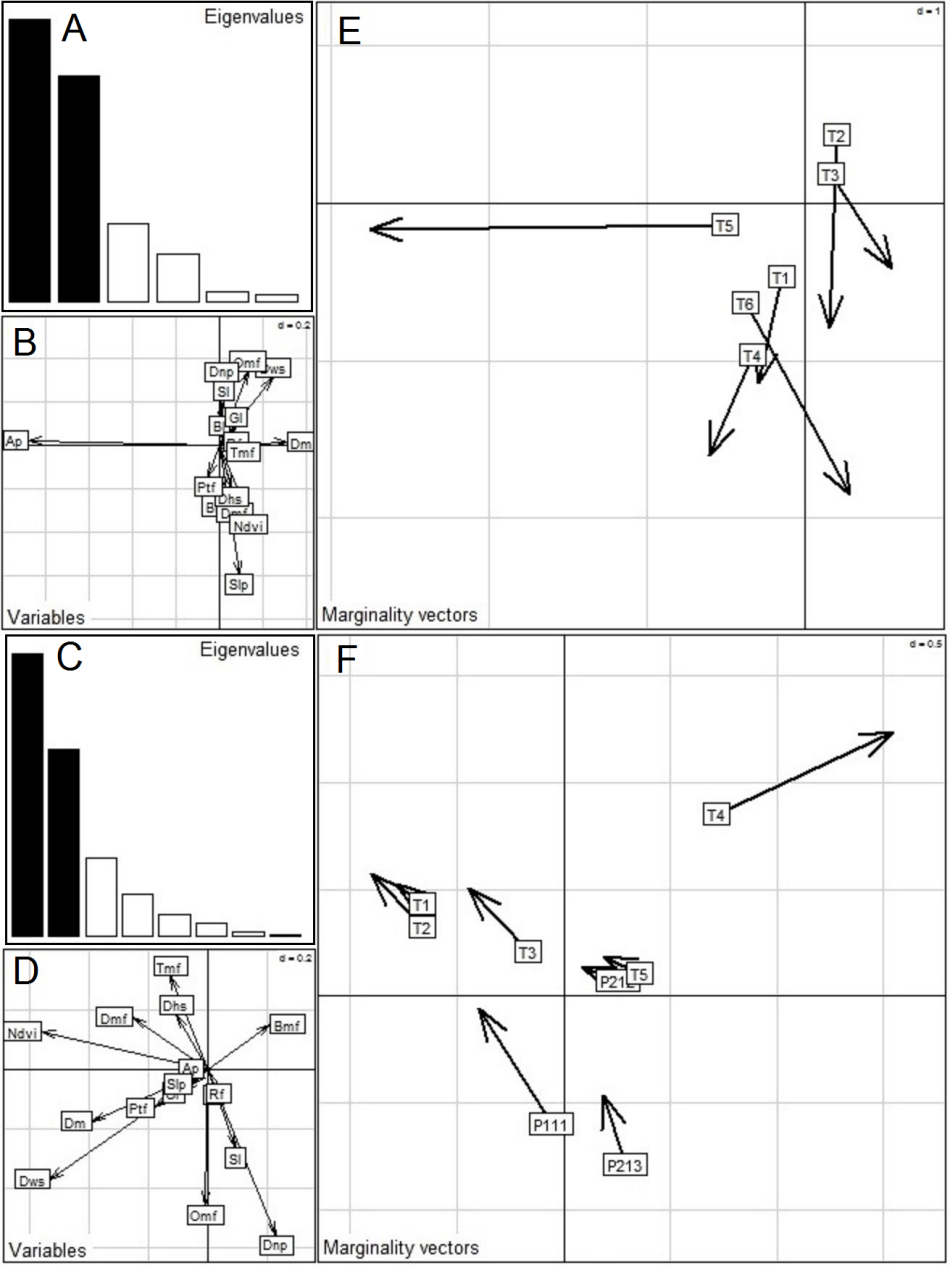
Results of K-select analysis carried out on 16 habitat variables to highlight habitat selection during the exploratory period and the settled period by 6 reintroduced tigers and 3 offspring. (B) and (D) are habitat variable loadings on first two factorial axes of the exploratory period and the settled period, respectively; (E) and (F) show the projection of marginality vectors of all animals on the first factorial plane during the exploratory and the settled periods, respectively. Both bar charts (A and C) of the eigenvalues show the mean marginality explained by each factorial axis. (Refer to Table 1 for detail descriptions of habitat variable abbreviations).

Habitat selection appeared significantly non-random for nearly all individuals in both the exploratory as well as the settled periods, as indicated by randomization tests carried out on marginality vectors (Table 2). The first eigenvalues for both the exploratory period (*λ*_1I_) and the settled period (*λ*_1S_) of this analysis were larger than expected under the random habitat use hypothesis (*λ*_1I_ = 0.89, *p* < 0.001 and *λ*_1S_ = 0.58, *p* < 0.001, respectively). In K-select analysis, bar plots of eigenvalues describe the magnitude of marginality explained by each factorial axis (Fig. 4). In the exploratory period, the first and second canonical axes of K-select analysis account for 43.7% and 34.9% of the total variance, respectively, while in the settled period these account for 46.2% and 30.5% of the total variance, respectively. Figures 4B and 4D provide scores of the predictor variables. During the exploratory period, T1 and T4 largely selected pure teak and bamboo mixed forest on steeper slopes, while also using riverine forest opportunistically. They avoided exploring areas outside the core area, scrub and barren lands. T2 largely used the elevated upper plateau of the reserve. T3 used highly vegetated areas in the National Park including dense mixed forest, teak mixed forest and bamboo mixed forest. T5 strongly selected *Anogeissus pendula* forest but opportunistically used riverine forest. T6 strongly selected highly vegetated areas such as teak mixed forest and dense mixed forests, again on complex terrain. All these tigers stayed close to major water sources such as river and man-made water bodies. During the settled period, habitat compositions in home range were markedly different from those occurring during the exploratory period. T1, T2, T3, T4, P111 and P213 used densely vegetated areas such as teak mixed forest, dense mixed forest and bamboo mixed forest. P212 used an elevated area as home range, while T5 continued to colonize a complex *Anogeissus pendula* forest.

### Habitat suitability map

**Figure 5:**
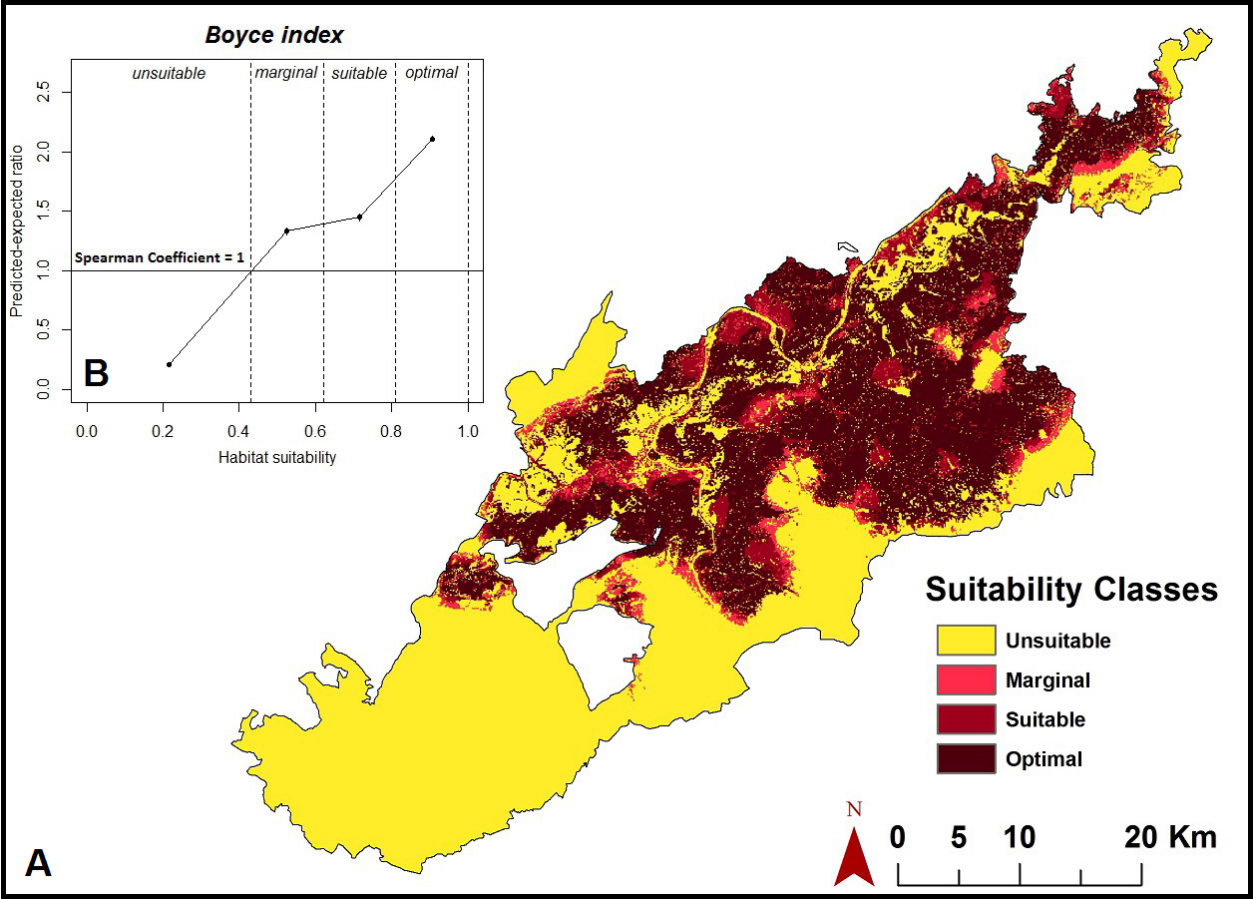
Habitat suitability map. (A) Map depicting habitat suitability for tigers in Panna Tiger Reserve, Madhya Pradesh, India. (B) shows predicted-to-expected ratios (*F*_*i*_ = 1) of evaluation points against 20 habitat suitability classes. The solid horizontal line (*F*_*i*_ = 1) is the curve of a random model, which was used as a threshold between unsuitable (*F*_*i*_ ≤ 1) and potential tiger (*F*_*i*_ > 1) habitats.

The habitat suitability map based on D^2^ statistics (Fig. 5, Fig. S1) clearly revealed that the habitats of all reintroduced tigers and their offspring largely fell in the core area of the tiger reserve. The suitable area extends in north-easterly and south-westerly directions on both sides of the core region. *Fi* values of the predictive map range between 0.2 and 2.2. The Boyce Index (Spearman correlation coefficient *r*: 1.00 and adjusted *r*^2^: 0.85) indicated a good predictive power of the suitability map. The map depicts that 47% of buffer and 83% of core region are potential tiger habitat in a range of marginal to optimal suitability. Of these, the central region of the park was predicted as most suitable for tigers, while peripheral areas mostly on the south-western side were least suitable (Fig. 5, Fig. S1).

## Discussion

The niche-based approach and Manly’s habitat selection ratio of type III design (Manly et al., 2002) enabled an understanding of habitat selection by tigers in dry deciduous forest. Our results include individual-level variation in habitat selection, which has not previously been empirically described for this species. We also present here for the first time fine-scale assessment of tiger habitat selection and spatially explicit modelling of habitat suitability based on long-term telemetry data. In a broader view, our results highlight the value of species restoration science towards understanding habitat ecology and behavioural biology of reintroduced species. Currently, Panna TR supports optimal densities of tiger prey species, distributed throughout the core area of the tiger reserve (Krishnamurthy et al., 2013). Although prey densities have a strong positive relationship with tiger densities (Karanth et al., 2004a; Miquelle et al., 2010), we did not include them in the analyses because prey densities are dynamic in response to predation pressure, climatic extremities, and poaching and these can affect the predictive ability of the model. The main focus of our analyses was to determine the habitat suitability based on EGVs so that habitat and population recovery measures can be designed. Also, very few habitat-oriented studies have demonstrated a relationship of tiger occurrence, distribution and habitat suitability to spatial distribution, and density of their prey species (Harihar & Pandav, 2012; Kawanishi & Sunquist, 2004; Sunarto et al., 2012). While the absence of density and spatial distribution data on prey species may appear to bring uncertainty in habitat prediction (Hebblewhite et al., 2012), the predictive model captures the fundamental characteristics and estimate of habitat availability, which is more important for recovery efforts.

Each tiger showed differences in habitat selection during exploratory as well as settled periods. However, the broad patterns are similar to other tiger studies such that tigers prefer dense vegetation (Johnsingh et al., 2004) but avoid areas with agricultural lands and human settlements (Smith, Ahern & McDougal, 1998; Karanth et al., 2004b; Carroll & Miquelle, 2006). Contrary to the inference of a study from Nepal that tigers and humans can coexist at fine spatial scales (Carter et al., 2012), our analysis demonstrates that habitat suitability at finer scales is founded on clear avoidance of human settlement and agricultural fields (Seidensticker, Christie & Jackson, 1999; Nyhus & Tilson, 2010; Krishnamurthy et al., 2016), although tigers may overlook anthropogenic correlates when undertaking long-distance dispersal, as demonstrated by another study from Panna TR (Krishnamurthy et al., 2016). It is reiterated that while the tiger may be tolerant of anthropogenic correlates at the landscape scale, specifically while undertaking dispersal, it clearly requires areas without human disturbance at fine scale. Therefore, the co-existence concept that is frequently discussed in the context of tiger conservation is scale-dependent and population-dependent.

The reintroduced tigers showed differential habitat choice between the exploratory and the settled periods. During the exploratory period, pure teak forest, *Anogeissus pendula* forest and water sources such as river and small wetlands were highly preferred by all reintroduced tigers. These land cover types (i.e., pure teak and *Anogeissus pendula* forests) are dominant near the Ken River and escarpments, and these areas provide a high level of natural protection and water. This was also the period when most of the tigers preferred riverine forests and water sources, especially man-made water bodies closer to village areas. Such association with human habitation during the exploratory period may possibly be due to unfamiliarity with resource distributions. The primary driver during such exploratory phase appears to be search for easy resources, overlooking disturbance elements. After settling, tigers preferred relatively undisturbed habitat with dense vegetation. The highly preferred vegetation includes riverine, teak mixed, *Anogeissus pendula* and dense mixed forest vegetation. Such habitats also harbour most of the major prey species such as spotted deer, sambar deer, wild pig, and Nilgai (Krishnamurthy et al., 2013). There was also noticeable variation in habitat preferences between both sexes of the reintroduced population. Females’ habitat preferences differed from males’ due to their exclusive requirement for undisturbed habitat and sufficient prey base for raising their young. Previous studies have demonstrated that female home range size is a function of available prey density (Smith, McDougal & Sunquist, 1987; Wilmers et al., 2013). Males, on the other hand, do not rely exclusively on food requirements and attempt to hold territories that encompass as many females as possible (Sandell, 1989). Because the tiger is a territorial species, individual-level behaviour responses also influence variation in habitat selection (Fraser et al., 2001; Bremner-Harrison, Prodohl & Elwood, 2004). This variability is an important consideration in habitat selection studies (Mysterud & Ims, 1998), particularly of populations that have been reintroduced (Sarkar et al., 2016; Bremner-Harrison, Prodohl & Elwood, 2004). Understanding individual habitat selection strategies may help managers to take certain critical decisions and interventions for the welfare of each reintroduced animal.

Suitability maps derived from tigers’ habitat preferences show that the core area of Panna TR has a high potential for harbouring tigers. Relocation of 13 villages from the core area also enabled restoration of most of the disturbed habitat. Vegetation in and around the vacated villages and cropland usually remain in the successional stage (Raman, Rawat & Johnsingh, 1998; Rangarajan & Shahabuddin, 2006) changing into pure grass or scrubland eventually. Scientific monitoring of relocated village lands within the National Park can be an option to improve nutritional quality of grass and plant species to support a wide range of ungulate species and also thwart unwanted weed invasion. Most of the newly created buffer region needs more attention towards improving habitat quality as our predicted suitability map shows >50% of newly proposed buffer area of TR is marginal or unsuitable for tigers, and the southwest part of buffer region will be avoided by tigers mainly due to presence of many villages and agriculture lands. Based on the fine-scale habitat selection knowledge generated in this study, management interventions will be required in these areas to transform the unsuitable habitats into effective buffers, so as to ensure long-term viability of the core region. Sex-specific behavioural responses, including habitat selection, can be used as a basis for managing other large carnivore species, particularly in cases involving reintroductions.

Taking a cue from the successful tiger recovery efforts in Panna TR, attempts are being made in other parts of India as well as in other countries. Feasibility assessment of such recovery efforts takes into consideration various ecological and anthropogenic parameters (Gray et al., 2017). However, it is always challenging to define the available habitat space for such recovery efforts since these low-density or locally extinct areas suffer from poaching of tiger and prey species. The modelling approach deployed in our study involving presence-only data, even from a small number of tigers or historical data, would yield a robust step forward for estimating habitat availability for recovery efforts. The sex-specific and scale-dependent choice for habitat also illustrate that the co-existence agenda is not uniformly applicable and has to be weighed against the spatial configuration of wild tiger population and anthropogenic correlates. Our results also provide an important lead for other tiger reserves to review the fine-scale occupancy of tiger populations and, accordingly, effect cost-effective management interventions towards immediate and long-term conservation benefits.

##  Supplemental Information

10.7717/peerj.3920/supp-1 Supplemental Information 1 Supporting data **Table S1:** Background information on the tigers and telemetry data used for fine scale-habitat selection analyses (F: female, M: male, W: wild, SW: Semi wild, a: GPS fixes, b: Location collected through VHF telemetry).

10.7717/peerj.3920/supp-2 Supplemental Information 2 Supporting data **Table S2:** Bivariate correlations of ecological and geographical variables used in K select analysis and habitat suitability modeling using Mahalanobis distance probability function. All values depict the Pearson correlation coefficients.
**Figure S1:** The continuous scale of habitat suitability map generated from Mahalanobish *D*^2^ method.

## Additional Information and Declarations

### Field Study Permissions

The following information was supplied relating to field study approvals (i.e., approving body and any reference numbers): Field experiments were approved by the Madhya Pradesh Forest Department, Madhya Pradesh, India.

### Data Availability

The following information was supplied regarding data availability: The raw data has been provided in the Supplemental Files.
