# Supplementary material for: Assessment of fine-scale resource selection and spatially explicit habitat suitability modelling for a re-introduced tiger (Panthera tigris) population in central India
Source: PeerJ. 2017 Nov 3;5:e3920. doi: 10.7717/peerj.3920 (PMC5672835; doi:10.7717/peerj.3920)
Supplement: Supplemental Information 1 — Table S1: Background information on the tigers and telemetry data used for fine scale-habitat selection analyses (F: female, M: male, W: wild, SW: Semi wild, a: GPS fixes, b: Location collected through VHF telemetry). [file peerj-05-3920-s001.docx]

**Table S1:** Basic information of reintroduced tigers and telemetry data used for habitat selection study (F: female, M: male, W: wild, SW: Semi wild, a: GPS fixes, b: Location collected through VHF telemetry)

| **Tiger (Sex)** | **Areal Distance (Km)** | **Age at 1^st^ Collaring**  **(Months)** | **Body Weight**  **(Kg)** | **Initial Exploration (Translocated individuals)** | | | **Initial Home Range (Founder and offspring)** | | |
| --- | --- | --- | --- | --- | --- | --- | --- | --- | --- |
|  |  |  |  | Duration | N _(Location)_ | Ẋ _(Location/day)_ ± SD | Duration | N _(Location)_ | Ẋ _(Location/day)_ ± SD |
| T1 (F-W) | 155 | 48 | 140 | 3^rd^ Mar 2009-15^th^ Aug 2009 | 1395^a^ | 9.52 ± 2.66 | 21^st^ Sep2011-14^th^ April 2012 | 526 ^a^ | 2.55 ± 2.88 |
| T2 (F-W) | 270 | 54 | 150 | 9^th^ Mar 2009-31^st^ Aug 2009 | 1380 ^a^ | 7.87 ± 2.33 | 23^rd^ Sep 2011- 10^th^ Aug 2012 | 562 ^a^ | 1.74 ± 2.44 |
| T3 (M-W) | 283 | 78 | 200 | 6^th^ Nov 2009 -31^st^ Jan 2010 | 557 ^a^ | 6.48 ± 1.41 | 1^st^ Sep 2011 - 29^th^ June 2012 | 560 ^a^ | 1.95 ± 2.73 |
| T4 (F-SW) | 270 | 54 | 106 | 26^th^ Mar 2011-31^st^ Aug 2011 | 1895 ^a^ | 11.99 ± 7.14 | 23^rd^ Jun 2012- 16^th^ Aug 2012 | 174 ^a^ | 3.16 ± 2.84 |
| T5 (F-SW) | 270 | 54 | 95 | 13^th^ Nov 2011-31^st^ Jan2012 | 635 ^b^ | 7.93 ± 6.84 | 1^st^ April 2012-23^rd^ July 2012 | 621^b^ | 5.40 ± 7.28 |
| T6 (F-W) | 280 | 48 | 105 | 23rd January 2015-31st March 2015 | 641 ^a^ | 9.42 ± 1.22 | - | - | - |
| P111 (M-W) | - | 21 | 130 | - | - | - | 6^th^ Jul 2012- 29^th^ Oct 2012 | 675 ^b^ | 5.87± 5.75 |
| P212 (M-W) | - | 19 | 110 | - | - | - | 6^th^ Aug 2012- 30^th^ Nov 2012 | 741^b^ | 6.68 ± 5.84 |
| P213 (F-W) | - | 19 | 87 | - | - | - | 1^st^ Aug 2012-31^st^ Oct 2012 | 660 ^b^ | 7.25± 6.18 |

S
