# Supplementary material for: Assessment of fine-scale resource selection and spatially explicit habitat suitability modelling for a re-introduced tiger (Panthera tigris) population in central India
Source: PeerJ. 2017 Nov 3;5:e3920. doi: 10.7717/peerj.3920 (PMC5672835; doi:10.7717/peerj.3920)
Supplement: Supplemental Information 2 — Table S2: Bivariate correlations of ecological and geographical variables used in K select analysis and habitat suitability modeling using Mahalanobis distance probability function. All values depict the Pearson correlation coefficients. Figure S1: The continuous scale of habitat suitability map generated from Mahalanobish D2 method. [file peerj-05-3920-s002.docx]

**Table S2:** Correlation among all ecological and geographical variables used in K select modelling and habitat suitability analysis using Mahalanobis distance probability function. All values depict the Pearson correlation coefficients.

|  | **Ana Pen** | **BamMxfor** | **Bar Lan** | **DEM** | **Den Mx For** | **Dis Hu Set** | **Dis Wat Sou** | **Gras Lan** | **NDVI** | **Open Mx For** | **Pur Tk For** | **Riv For** | **Scr Lan** | **Slope** | **Tk Mx For** | **DistNP** |
| --- | --- | --- | --- | --- | --- | --- | --- | --- | --- | --- | --- | --- | --- | --- | --- | --- |
| **Ana Pen** | 1.00 |  |  |  |  |  |  |  |  |  |  |  |  |  |  |  |
| **Bam For** | - 0.01 | 1.00 |  |  |  |  |  |  |  |  |  |  |  |  |  |  |
| **Bar Lan** | - 0.02 | - 0.02 | 1.00 |  |  |  |  |  |  |  |  |  |  |  |  |  |
| **DEM** | - 0.12 | - 0.04 | - 0.07 | 1.00 |  |  |  |  |  |  |  |  |  |  |  |  |
| **Den Mx For** | - 0.05 | - 0.07 | - 0.14 | 0.01 | 1.00 |  |  |  |  |  |  |  |  |  |  |  |
| **Dis Hu Set** | 0.01 | 0.05 | 0.24 | - 0.11 | 0.03 | 1.00 |  |  |  |  |  |  |  |  |  |  |
| **Dis Wat Sou** | - 0.09 | - 0.04 | 0.17 | 0.51 | - 0.12 | - 0.13 | 1.00 |  |  |  |  |  |  |  |  |  |
| **Gras Lan** | 0.00 | - 0.01 | - 0.01 | - 0.03 | - 0.03 | 0.02 | - 0.03 | 1.00 |  |  |  |  |  |  |  |  |
| **NDVI** | 0.02 | 0.20 | - 0.24 | 0.01 | 0.33 | - 0.02 | - 0.21 | - 0.03 | 1.00 |  |  |  |  |  |  |  |
| **Open Mx For** | - 0.06 | - 0.06 | - 0.14 | 0.23 | - 0.32 | 0.00 | 0.09 | - 0.03 | - 0.03 | 1.00 |  |  |  |  |  |  |
| **Pur Tk For** | - 0.01 | - 0.01 | - 0.02 | - 0.18 | - 0.07 | - 0.05 | - 0.08 | 0.00 | 0.07 | - 0.07 | 1.00 |  |  |  |  |  |
| **Riv For** | - 0.01 | - 0.01 | - 0.01 | - 0.12 | - 0.04 | - 0.01 | - 0.10 | 0.00 | - 0.10 | - 0.04 | - 0.01 | 1.00 |  |  |  |  |
| **Scr Lan** | - 0.04 | - 0.04 | - 0.08 | - 0.08 | - 0.24 | 0.30 | 0.10 | - 0.02 | - 0.30 | - 0.24 | - 0.04 | - 0.03 | 1.00 |  |  |  |
| **Slope** | 0.04 | 0.14 | - 0.11 | - 0.09 | 0.33 | 0.14 | - 0.15 | - 0.01 | 0.29 | - 0.11 | 0.03 | 0.00 | - 0.13 | 1.00 |  |  |
| **Tk Mx For** | - 0.02 | - 0.02 | - 0.05 | - 0.02 | - 0.13 | - 0.02 | - 0.11 | - 0.01 | 0.33 | - 0.16 | - 0.03 | - 0.02 | -0.10 | 0.01 | 1.00 |  |
| **Dist NP** | - 0.09 | - 0.03 | 0.16 | 0.35 | - 0.17 | - 0.14 | 0.87 | - 0.05 | - 0.27 | 0.04 | - 0.07 | - 0.05 | 0.14 | -0.14 | -0.13 | 1.00 |


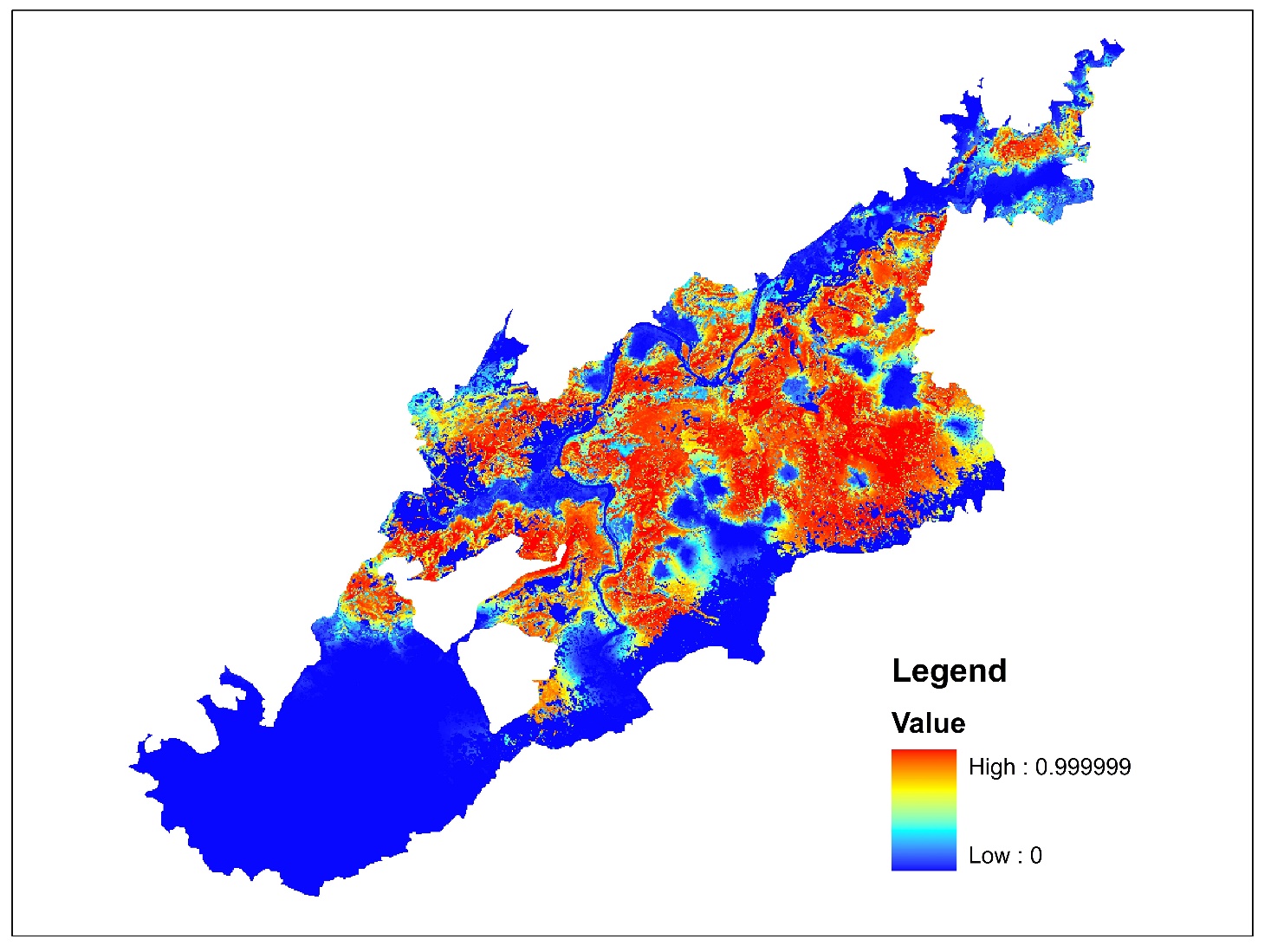


**Figure S1:** The continuous scale of habitat suitability map generated from Mahalanobish D^2^ method
